# Phylogeny, Floral Evolution, and Inter-Island Dispersal in Hawaiian *Clermontia* (Campanulaceae) Based on ISSR Variation and Plastid Spacer Sequences

**DOI:** 10.1371/journal.pone.0062566

**Published:** 2013-05-02

**Authors:** Thomas J. Givnish, Gregory J. Bean, Mercedes Ames, Stephanie P. Lyon, Kenneth J. Sytsma

**Affiliations:** 1 Department of Botany, University of Wisconsin-Madison, Madison, Wisconsin, United States of America; 2 Chesterfield Village Research Center, Monsanto, Chesterfield Village, Missouri, United States of America; 3 Madison Campus, Promega North America, Fitchburg, Wisconsin, United States of America; Instituto de Higiene e Medicina Tropical, Portugal

## Abstract

Previous studies based on DNA restriction-site and sequence variation have shown that the Hawaiian lobeliads are monophyletic and that the two largest genera, *Cyanea* and *Clermontia*, diverged from each other ca. 9.7 Mya. Sequence divergence among species of *Clermontia* is quite limited, however, and extensive hybridization is suspected, which has interfered with production of a well-resolved molecular phylogeny for the genus. *Clermontia* is of considerable interest because several species posses petal-like sepals, raising the question of whether such a homeotic mutation has arisen once or several times. In addition, morphological and molecular studies have implied different patterns of inter-island dispersal within the genus. Here we use nuclear ISSRs (inter-simple sequence repeat polymorphisms) and five plastid non-coding sequences to derive biparental and maternal phylogenies for *Clermontia*. Our findings imply that (1) *Clermontia* is not monophyletic, with *Cl. pyrularia* nested within *Cyanea* and apparently an intergeneric hybrid; (2) the earliest divergent clades within *Clermontia* are native to Kauài, then Òahu, then Maui, supporting the progression rule of dispersal down the chain toward progressively younger islands, although that rule is violated in later-evolving taxa in the ISSR tree; (3) almost no sequence divergence among several *Clermontia* species in 4.5 kb of rapidly evolving plastid DNA; (4) several apparent cases of hybridization/introgression or incomplete lineage sorting (i.e., *Cl. oblongifolia, peleana, persicifolia, pyrularia, samuelii, tuberculata*), based on extensive conflict between the ISSR and plastid phylogenies; and (5) two origins and two losses of petaloid sepals, or—perhaps more plausibly—a single origin and two losses of this homeotic mutation, with its introgression into *Cl. persicifolia.* Our phylogenies are better resolved and geographically more informative than others based on ITS and 5S-NTS sequences and nuclear SNPs, but agree with them in supporting *Clermontia's* origin on Kauài or some older island and dispersal down the chain subsequently.

## Introduction

The Hawaiian lobeliads (Campanulales: Campanulaceae) are the largest family of flowering plants (6 genera, ca. 130 species) native to the Hawaiian Islands, and have undergone specta cular adaptive radiations in growth habit, floral morphology, habitat, leaf form, and photosynthetic physiology [1]–[16]. Recent analyses based on DNA sequence variation indicate that this group is the product of a single colonization event about 13 million years ago (Mya), and that the two largest genera – *Cyanea* (ca. 80 spp.) and *Clermontia* (22 spp.) – diverged from each other roughly 9.7 Mya near the now eroded seamounts of the French Frigate Shoals and Gardner Pinnacles, long before the oldest tall island today (Kauài) emerged above the Pacific 4.7 Mya [14]. Previous research on *Cyanea*, the largest genus of flowering plants native to Hawaii, produced a partial phylogeny based on plastid DNA restriction-site variation which showed a strong relationship between geographic distribution and ancestry [8], [9]. Most of the dispersal events implied by that tree were from one island to the next, younger island in the Hawaiian chain, supporting the so-called “progression rule” [17], with most of the inferred inter-island dispersal events having been from one island to the next younger, ecologically unsaturated one in the chain, as expected from ecological theory [8], [9].

Extant *Cyanea* species began diversifying soon after the genus split from *Clermontia*, whereas extant *Clermontia* began diverging from each other only in the last 5 My [14]. As a consequence, genetic divergence among species of *Clermontia* is quite limited, and it has proven difficult to derive a phylogeny for *Clermontia* based on either morphological or molecular variation. Lammers [6] derived a moderately resolved phylogeny of *Clermontia* based on a maximum-parsimony analysis of morphological characters, and used that phylogeny to infer the origin of the genus (or at least the extant lineage) on the youngest island of Hawaìi, which emerged from the Pacific no more than 0.5 Mya, and to imply several dispersal events back up the Hawaiian chain to older islands. This contrasts with the conclusion by Givnish et al. [9], [14] –based on plastid restriction and sequence data, though with few species of *Clermontia* included – that *Clermontia* and its sister genus *Cyanea* arose ca. 9.7 Mya, on islands now reduced to pinnacles and reefs, with most dispersal events down the chain to younger islands. However, six of Lammers' 32 characters – including sepal size, shape, color, texture, fusion, and persistence – hinge simply on whether the sepals are like or unlike the petals. Hofer et al. [18] recently proposed that petaloid sepals in *Clermontia* represent a homeotic mutation, involving the ectopic expression of *PISTILLATA* B-function MADS box gene homologs in the outermost floral whorl, and present gene-expression data supporting their view. Given this, Lammers' six sepal characters should be coded as only one – petaloid sepals present or absent. When we re-analyze Lammers' data making this change, almost all resolution disappears in the unweighted strict consensus tree, including the distinction between the two traditional sections based on sepaloid petals being present (sect. *Clermontia*) vs. absent (sect. *Clermontioides*) (see **Results**). Hofer et al. [18] presented a molecular phylogeny for *Clermontia* based on cloned sequences of the non-transcribed spacer of 5S rDNA. Although poorly resolved, their tree does place the Kauài endemic *Clermontia fauriei* sister to all other *Clermontia* species, consistent with an origin for the genus on Kauài or some older island. Pillon et al. [19] also present a poorly resolved phylogeny for *Clermontia,* based on 31 single-nucleotide polymorphisms (SNPs) for >200 accessions; their analysis places *Cl. fauriei* sister to all other members of the genus except *Cl. pyrularia,* which is instead resolved as part of *Cyanea*. Several *Clermontia* hybrids of recent origin have been suspected based on their having a morphology intermediate to that of the putative parental taxa, including apparent crosses involving *Cl. calophylla, Cl. drepanomorpha, Cl. hawaiiensis, Cl. kohalae, Cl. montis-loa, and Cl. parviflora* on Hawaìi, as well as between *Cl. kakeana and Cl. micrantha* on Maui [1], [2], [5], [6], [7].

Studies of evolution within *Clermontia* have been stymied by a lack of a reliable, well-resolved phylogeny based on markers that evolve with sufficient rapidity. The lack of comparisons between trees for *Clermontia* based on maternally inherited plastid markers and biparentally inherited nuclear markers has similarly hampered progress toward identifying and understanding instances of potentially widespread reticulate evolution within the genus. Here we present data from rapidly evolving, predominantly nuclear ISSRs (inter-simple sequence repeat polymorphisms) and five plastid spacer or intron sequences, and employ them to create biparental and maternal phylogenies for *Clermontia*, respectively. We then use these trees to test the monophyly of *Clermontia,* evaluate the extent of genetic divergence within the genus, infer the pattern of inter-island dispersal during its evolution, reconstruct potential cases of reticulate evolution, and identify the number and timing of origins and losses of the apparent homeotic mutation for petaloid sepals.

## Methods

### Phylogenetic analysis of morphological characters

We re-scored Lammers' morphological dataset (32 characters × 25 taxa, including one generalized *Cyanea* and two geographic populations each for *Cl. oblongifolia* and *Cl. peleana*) for *Clermontia* [6] by excluding five of the six characters representing the presence/absence of petaloid sepals, retaining sepal texture. The remaining 27 characters were then re-analyzed using maximum parsimony (MP) in PAUP* 4.0b8 [20]. We initiated 10,000 replicate MP searches with random starting trees and used TBR branch-swapping. This approach increased the chance of detecting multiple islands of equally parsimonious trees. We formed the strict consensus of all shortest trees recovered. Bootstrap support for each node was estimated using 1000 random resamplings of the data, with each replicate search seeded by a new random-addition tree. Given the few morphological characters, we also used successive reweighting of characters based on the consistency index (CI) of each character [21], [22], and recalculated the strict consensus tree and bootstrap values for each node using the reweighted data.

### Phylogenetic analysis of ISSR and cpDNA characters

Genomic DNAs used in this study were collected and extracted for previous analyses [8], [9]. Thirty-four accessions representing all 21 extant species of *Clermontia* were surveyed, along with two outgroups consisting species of the sister genus *Cyanea* (*Cy. leptostegia* and *Cy. pilosa* ssp. *longipedunculata,* representing the purple- and orange-fruited clades [8], [9]). For each species of *Clermontia* which occurs on more than one island, or in more than one biogeographic region within an island (e.g., East vs. West Òahu), samples from each island and biogeographic region were included whenever possible. Table 1 lists the populations represented; nomenclature follows Lammers [7]. Thirty-two accessions were included in the ISSR survey; 31, in the plastid sequence study (Table 1).

**Table 1:** Taxa included in study, with geographic distribution, voucher data, and GenBank accession numbers for plastid sequences.

Taxon and distribution*	Voucher data	*rpl16* intron	*trnL-trnF*	*trnT-trnL*	*trnV-trnK*	*atpB-rbcL*
*Clermontia arborescens* Mo	Smith et al. 1162, WIS	DQ285102	DQ285141	DQ285219	DQ285180	DQ285258
*Cl. arborescens* L	Smith 1127, WIS	KC460601	KC460628	KC460576	KC469899	KC460546
*Cl. arborescens* WM	Smith et al. 1165, WIS	KC460600	KC460630	KC460573	KC469901	KC460549
*Cl. arborescens* EM	Smith 2193, WIS	KC460602	KC460627	KC460574	KC469898	KC460547
*Cl. arborescens* Mo	Smith et al. 1159, WIS	KC460603	KC460629	KC460575	KC469900	KC460548
*Cl. calophylla* H	Smith 2136, WIS	KC460604	KC460631	KC460577	KC469902	KC460550
*Cl. clermontioides* H	Sytsma 5099, WIS	KC460605	KC460632	KC460578	KC469903	KC460551
*Cl. drepanomorpha* H	Smith 2140, WIS	KC460606	KC460633	KC460579	KC469904	KC460552
*Cl. fauriei* K	Smith et al. 1137, WIS	DQ285103	DQ285142	DQ285220	DQ285181	DQ285259
*Cl. grandiflora* L	Smith 1130, WIS	KC460607	KC460634	KC460580	KC469905	KC460554
*Cl. grandiflora* WM	Smith et al. 1166, WIS	KC460608	KC460635	KC460581	KC469906	KC460553
*Cl. grandiflora* EM	Sytsma 5083, WIS					
*Cl. hawaiiensis* H	Givnish s.n., WIS	KC460609	KC460636	KC460582	KC469907	KC460555
*Cl. kakeana* WO	Smith 2146, WIS	KC460610	KC460640	KC460583	KC469911	KC460558
*Cl. kakeana* Mo	Givnish s.n., WIS	KC460613	KC460638	KC460586	KC469909	KC460557
*Cl. kakeana* WM	Givnish & Sytsma 3003, WIS	DQ285104	DQ285143	DQ285221	DQ285182	DQ285260
*Cl. kakeana* WM	Sytsma, Smith, Givnish 5078, WIS	KC460611	KC460639	KC460585	KC469910	KC460559
Cl. *kakeana* EM	Smith 2201, WIS	KC460612	KC460637	KC460584	KC469908	KC460556
*Cl. kohalae* H	Alverson 2200, WIS	KC460614	KC460641	KC460587	KC469912	KC460560
*Cl. lindseyana* EM	Smith 2202, WIS	KC460615	KC460642	KC460589	KC469913	KC460561
*Cl. lindseyana* H	Givnish 2211, WIS	KC460616	KC460643	KC460588	KC469914	KC460562
*Cl. micrantha* WM	Sytsma, Smith, Givnish 5069, WIS					
*Cl. montis-loa* H	Givnish s.n., WIS	KC460617	KC460644	KC460590	KC469915	KC460563
*Cl. oblongifolia* WO	Smith 2134, WIS	KC460618	KC460645	KC460591	KC469916	KC460564
*Cl. oblongifolia* EO	J. Obata, field id					
*Cl. pallida* Mo	Smith et al. 1161, WIS	KC460619	KC460646	KC460592	KC469917	KC460565
*Cl. parviflora* H	Smith 2142, WIS	DQ285132	DQ285171	DQ285249	DQ285210	DQ285288
*Cl. peleana* H	Lyon Arboretum, PO169	KC460620	KC460647	KC460593	KC469918	KC460566
*Cl. persicifolia* WO	Smith 2212, WIS	KC460621	KC460649	KC460594	KC469920	KC460567
*Cl. persicifolia* EO	Sytsma, Smith, Obata 5016, WIS	KC460622	KC460648	KC460595	KC469919	KC460568
*Cl. pyrularia* H	L. Cuddihy, field id	KC460623	KC460650	KC460596	KC469921	KC460569
*Cl. samuelii* EM	Sytsma, Smith, Givnish 5071, WIS	KC460624	KC460651	KC460597	KC469922	KC460570
*Cl. tuberculata* EM	Sytsma, Smith, Givnish 5070, WIS	KC460625	KC460652	KC460598	KC469923	KC460571
*Cl. waimeae* H	Smith 2152, WIS	KC460626	KC460653	KC460599	KC469924	KC460572
*Cyanea leptostegia* K	Smith et al. 1135, WIS	DQ285133	DQ285172	DQ285250	DQ285211	DQ285289
*Cyanea pilosa* ssp. *longipedunculata* H	Givnish 3105, WIS	DQ285135	DQ285174	DQ285252	DQ285213	DQ285291

Taxa without GenBank numbers were analyzed for ISSR variation only.

#### ISSR variation

The ISSR technique is based on surveying variation in the length of the spacers between short simple repeats (SSRs) of a particular kind [23], [24]. In practice, this was achieved by using PCR primers that anneal to a specific SSR to amplify the intervening spacers. After amplification, PCR products were size-separated by electrophoresis; bands of ISSR DNA of different lengths were visualized by staining the gel with ethidium bromide and photographing it on a UV light table. Different taxa were scored by running ISSRs generated by a given primer pair side-by-side on the same gel, and then tallying the presence/absence of different bands across taxa.

We screened 58 different 2- and 3-base-pair repeat primers for their effectiveness in producing useful, repeatable, interpretable levels of variation ISSR length in four taxa (*Clermontia calophylla* [N Hawaii], *C. lindseyana* [E Maui], *C. parviflora* [W Hawaii], and *C. tuberculata* [E Maui]), which Lammers [5] classified as members of different sections and series. Primers were drawn from sets provided by the University of British Columbia (Vancouver, BC) and Integrated DNA Technologies, Inc. (Coralville, IA). Nine primers (Table 2) were chosen to survey ISSR variation across populations because they produced repeatable band polymorphisms, involving a large number of co-migrating bands that were easily scorable by eye. Given the relative sizes of the nuclear genome of Hawaiian lobeliads (4C  =  4106 Mb for *Brighamia insignis,* Kew C-value database, http://data.kew.org/cvalues/) vs. that of the plastid genome (ca. 0.15 Mb), essentially all ISSR bands should reflect variation in nuclear DNA.

**Table 2:** ISSR primers used in this study.

1	TCT	CTC	TCT	CTC	TCT	CG
2	AGA	GAG	AGA	GAG	AGA	GYT
3	GAG	AGA	GAG	AGA	GAG	AYT
4	GAG	AGA	GAG	AGA	GAG	AYG
5	CTC	TCT	CTC	TCT	CTC	TRA
6	CAC	ACA	CAC	ACA	CAC	ART
7	CAC	ACA	CAC	ACA	CAC	ARG
8	CTC	TCT	CTC	TCT	CTC	TRG
9	CAC	ACA	CAC	ACA	RY	

Y  =  C or T, R  =  A or G.

We used a slight modification of the Tsumara et al. [25] protocol for ISSR amplification. PCR reactions consisted of 28.5 µl double-distilled H_2_O, 4.0 µl 25 mM MgCl_2_, 5.0 µl 10X Mg-free reaction buffer, 8.0 µl 1.25 mM dNTPs, 2.5 µl 4 mg/ml BSA, 1.0 µl formamide, 0.5 µl 20 M primer, 0.25 µl *Taq* polymerase, and 0.5 µl template DNA. PCR was conducted using a Perkin-Elmer Gene Amp 2400 thermal cycler, using the following program: initial denaturation of 7 min at 94°C; then 45 cycles of 30 s at 94°C, 45 s at 52°C, and 120 s at 72°C; and a final extension period of 7 min at 72°C. Amplified products were size-separated on 2% agarose gels using 0.5X TBE buffer, stained with ethidium bromide, photographed on a UV light table, and visually scored for the presence/absence of individual bands.

Questions have arisen from time to time about the possible lack of homology of comigrating bands associated with ISSR markers and similarly produced RAPDs and AFLPs and the potential impact of such noise on phylogenetic inference [26]–[31]. In general, however, many investigators have found that phylogenies based on such arbitrarily amplified markers are highly concordant with, but better resolved than those based on plastid or ITS sequences and/or restriction sites ([29], [31]–[36] for AFLPs, [28], [37] for RAPDs, [38]–[40] for ISSRs). Rieseberg [27] found that 91% of 220 RAPD markers used to study interspecific relationships in *Helianthus* appeared to reflect homologous markers; Ipek et al. [30] found that 95% of AFLP amplicons in garlic were identical or highly homologous to the typical marker for that band. In general, the likelihood of homoplasy among AFLP, RAPD, or ISSR fragments should increase with genetic distance of the species studied, so that they should be useful for phylogenetic inference if sequence divergence among the ingroup taxa is low [29], [41], [42]. Indeed fragment-based markers have been successfully to reconstruct phylogenies for a number of recent and rapidly radiating groups [29], [31], [33], [35], [38]–[48], including implementation of an AFLP clock in at least one case [46].

#### Plastid sequence data

DNA samples were sequenced for four rapidly evolving plastid intergenic spacers – *atpB-rbcL, trnL-trnF, trnT-trnL, trnV-trnK* – and the *rpl16* intron following Givnish et al. [14]. Three taxa surveyed for ISSR variation (*Cl. grandiflora* from East Maui, *Cl. micrantha,* and *Cl. oblongifolia* from East Òahu) were not sequenced for lack of DNA, leaving a total of 31 accessions. All new sequences were uploaded to GenBank and accession numbers obtained (Table 1).

#### Phylogenetic analyses

ISSR and plastid sequence data were analyzed independently using MP in PAUP*, using the same approach as for morphological data (see above), employing *Cyanea leptostegia* and *Cy. pilosa* ssp. *longipedunculata* as outgroups, and using unweighted and (for ISSR data) successively weighted analyses to calculate strict consensus trees and bootstrap values. Plastid sequence data were also analyzed using Bayesian inference (BI) and maximum likelihood (ML). BI was implemented in MrBayes 3.2 [49], using a model of sequence evolution (GTR + G + I model) that best fit the entire concatenated plastid data identified with jModelTest [50]. Posterior probabilities were approximated in a search over 4,000,000 generations via four simultaneous MCMC chains with every 1,000^th^ tree saved. Default values were used for MCMC parameters. Of the resulting trees the first 20% (burn in) were discarded. The remaining trees were summarized in a majority rule consensus tree yielding the probabilities for each clade to be monophyletic. To ensure that the MCMC chains explored total tree space, we replicated these runs with four different random starting trees. ML used Garli 2.0 [51] implemented in the Cyberinfrastructure for Phylogenetic Research (CIPRES) portal 2 teragrid [52]. Maximum-likelihood bootstrapping was executed using RAxML 7.0.4 [53], [54].

To assess conflict in phylogenetic structure between the unweighted ISSR and plastid data, we conducted an incongruence length difference (ILD) test [55] in PAUP*. It is widely recognized that the ILD test has several limitations [56], [57], but is can be useful to identify broad-scale incongruence between datasets. Incongruence was also evaluated based on the presence of moderately supported (≥ 70% BS value) differences between nodes of the plastid and ISSR phylogenies. Incongruence between maternally inherited vs. biparentally inherited markers can reflect differences in their evolutionary history, reflecting hybridization, introgression, or chloroplast capture. To the extent that incongruence reflects such processes, plastid and nuclear data should not be combined. If there is no significant conflict, concatenating the data sets can improve their power to resolve relationships.

We utilized the following steps to assess the degree of incongruence between plastid sequence and ISSR data. First, taxa not scored for both data sets were removed; second, missing plastid sequences for one population each of *Cl. oblongifolia* and *Cl. grandifolia* were replaced with those from another population of each of the species from the same island. If these data sets then showed significant incongruence, we pruned taxa whose position on the ISSR and plastid sequence trees conflicted with bootstrap support ≥ 70%. We also pruned the single taxon (*Cl. pyrularia*) placed by morphology in *Clermontia* but by molecular data in *Cyanea*. We then re-analyzed the pruned data sets separately and pruned any additional taxa whose position on the ISSR and plastid trees conflicted with BS ≥ 70%. We combined the pruned ISSR and plastid data sets if they did not then exhibit significant incongruence under the ILD test, and analyzed the joint data using MP and BI. For BI analyses in MrBayes, the binary ISSR data were included as a separate partition of sequence characters with only that partition evaluated under the simplistic Jukes-Cantor model. The combined data set was also analyzed using BI in BEAST [58], [59] as described below.

### Historical biogeography

Ancestral area reconstruction (AAR) and between-island dispersal events were analyzed using the contrasting methods and accompanying assumptions implemented in BEAST [58], [59], DEC [60], MacClade [61], and BayesTraits [62]. We categorized the eight current tall islands of the Hawaiian chain into four groups, reflecting terrestrial connections during sea-level lows during the Pleistocene: (i) Kauài and Nìihau; (ii) Òahu; (iii) Molokài, Lanài, Maui, and Kahòolawe; and (ii) Hawaìi. Islands within group (i) and (iii) were interconnected in the recent past, with connections having been lost partly due to island subsidence and cyclical sea-level change. Pleistocene glacial cycles lowered ocean levels by as much as 120 m, resulting in the islands of Maui Nui (group iii) being connected as recently as last glacial period, and having been interconnected with each other for about 75% of the last million years [63].

In order to obtain a time-calibrated phylogenetic tree for *Clermontia*, we used a relaxed clock approach as implemented in BEAST v1.7.4 with the uncorrelated lognormal model on the combined, taxon-reduced data set. The two data sets were brought in as separate partitions. The “simple substitution model” was used for the binary ISSR data partition, whereas the full GTR substition model was used for the plastid data partition. We explored both the Yule process and the birth-death speciation tree priors. Trial runs were used to determine the number of generations necessary to achieve an effective sample size (ESS) of at least 200 in Tracer v1.5, and to optimize the operator settings for our final analyses. For each of the two speciation tree priors, we ran 15 million generations on two computers, each starting with a randomly generated tree. Samples were taken every 1,000 generations, and the first 1.5 million generations of each run were discarded as burn-in. The resulting 13,500 trees from each run were combined with LogCombiner v1.6.1. The trees were then interpreted by TreeAnnotator v1.6.1 prior to visualization in FigTree v1.3.1. We constrained three nodes of *Clermontia* and *Cyanea* with normal priors using stem and crown ages of *Clermontia* and *Cyanea* derived from the Hawaiian lobeliad phylogeny based on sequences of eight plastid spacers and calibrated with asterid fossils [14]. The root of *Clermontia* + *Cyanea*, the crown of *Cyanea*, and the crown of *Clermontia* were set at 9.74, 9.58, and 4.33 Mya, respectively, and with standard deviations to permit age ranges matching the 95% confidence intervals in the Hawaiian lobeliad chronogram [14]. We also constrained the nodal ages of two clades that radiated exclusively on Hawaìi and Maui Nui, respectively, based of post-emergence ages of the two islands of 0.6 ± 0.1 Mya and 2.0 ± 0.1 Mya, respectively [64]. Additionally, the MP tree (see above) was converted to a chronogram in BEAST by inputting the tree as a start tree and not allowing the topology to change by removing all the operators that act on the treemodel (narrowExchange, wideExchange, wilsonBalding, and subtreeSlide).

MP reconstructions of geographic distribution used MacClade applied to the MP and BI chronograms based on the combined molecular data. We assumed a basal condition for *Cyanea* of Kauài or some older, former tall island, following Givnish et al. [9] and the estimated stem age of *Clermontia* and *Cyanea* being 9.74 Mya, long before any of the current tall islands rose above the Pacific [14]. We used the resolving option of “all most parsimoniuous states at each node” for the initial mapping, and then used delayed transformation (DELTRAN) to produce a wholly resolved set of transitions and a map summarizing them, based on the conservative assumption that inter-island dispersal is an unlikely phenomenon and thus should be assumed only when absolutely necessary. In practice, but not through constraint, DELTRAN created the same resolution of dispersal events as would have a resolution of any uncertainties favoring the progression rule. Colonization and dispersal across the Hawaiian Islands were also reconstructed under BI criteria using the BayesMultiState option in BayesTraits applied to the combined molecular tree. To reduce some of the uncertainty and arbitrariness of choosing priors under MCMC, we used the recommended hyperprior approach (rjhp command) [62]. We used a random subset of 100 post burn-in Bayesian trees (Perl script from [65]).

We implemented a DEC (dispersal-extinction-cladogenesis) analysis in Lagrange following the method used with Hawaiian *Psychotria* [60]. We modeled dispersal across the Hawaiian chain by employing five time intervals (> 5.1 Mya for “pre-Kauài”, 5.1–3.9 Mya for Kauài, 3.9–2.2 Mya for O'ahu [66], 2.2–0.7 for Maui Nui, and < 0.7 Mya for Hawaìi). In each time interval the possibility of forward dispersal to islands presently above water was 1.0, and 0.0 to all other islands. Backward dispersal to older islands was permitted, but at a reduced probability of 0.1. The adjacency matrix was restricted to neighboring islands, although this configuration had no impact on the results. The BEAST tree derived from both molecular data sets was used as the basis for the DEC analysis.

### Petaloid sepal character-state reconstruction

The presence/absence of petaloid sepals was overlaid on the MP and BI chronograms based on the combined molecular data using MP implemented in MacClade [37], with the resolving option of “all most parsimoniuous states at each node”. BI overlays were assembled using MultiState and a random set of 100 Bayesian PP trees in BayesTraits [38]. Ancestral reconstruction of character evolution under BI with the 100 random PP trees was represented by pie charts indicating state probabilities at each node in the combined DNA chronogram.

## Results

### Morphology-based phylogeny

**Figure 1:**
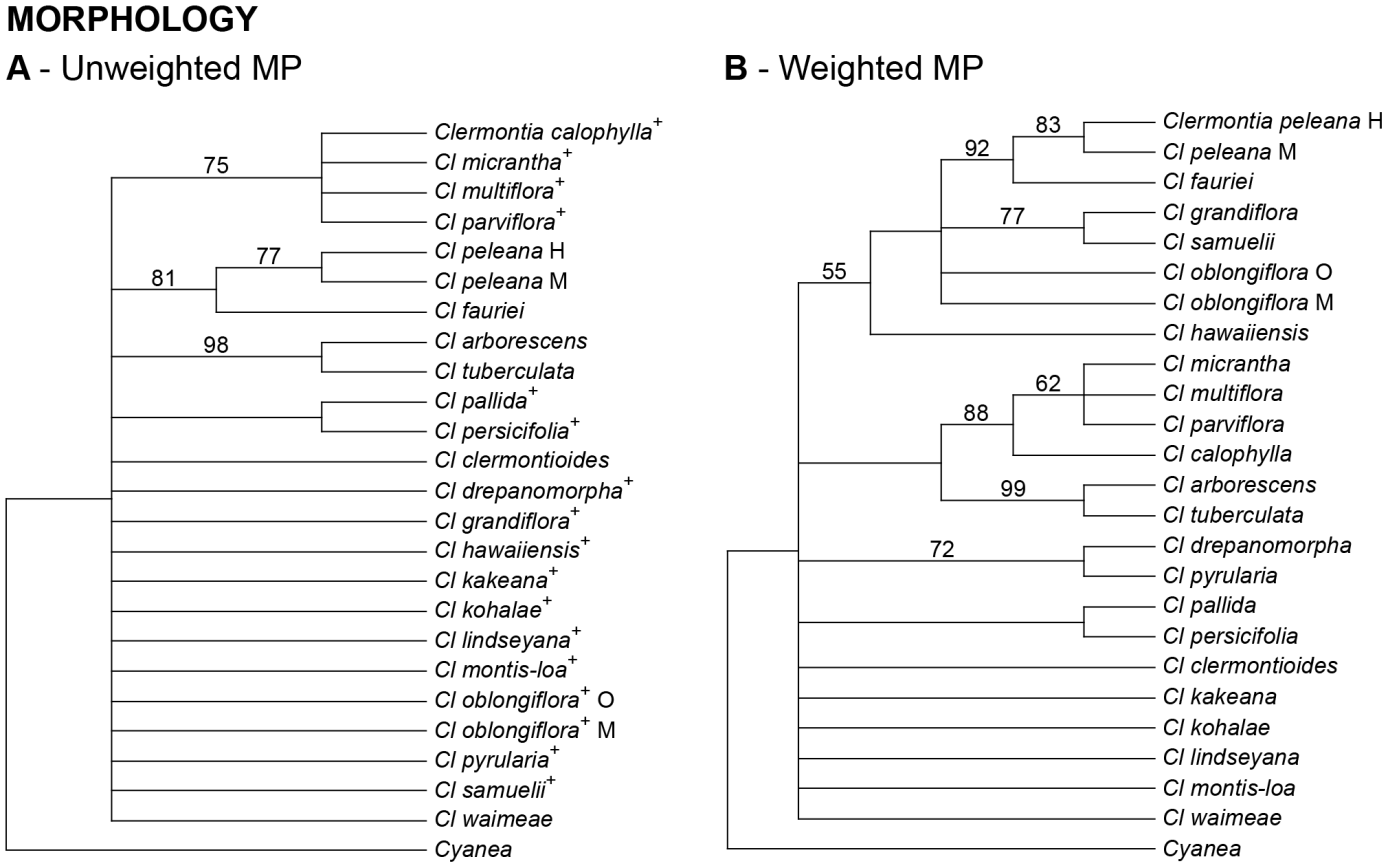
Phylogenetic relationships within *Clermontia* (24 taxa and 1 outgroup *Cyanea*) based on 27 morphological characters, scoring petaloid sepals as a single state of a single character. Numbers above branches indicate bootstrap support levels ≥ 20%. (A) Maximum-parsimony (MP) phylogeny based on unweighted characters. The tree shown is strict consensus of 4928 shortest trees. Superscript + indicates presence of petaloid sepals. (B) MP phylogeny based on sequential reweighting of morphological characters; tree shown is strict consensus of 100 shortest trees.

The morphological data matrix yielded one island of 4928 shortest trees, each 49 steps long (CI  =  0.69; CI'  =  0.61 excluding autapomorphies), and a strict consensus phylogeny that resolved only 5 of 22 potential nodes within *Clermontia* (**Fig. 1A**). Seventeen of 27 characters were parsimony-informative. Unweighted morphological variation grouped together three pairs of species (*arborescens-tuberculata, fauriei-peleana,* and *pallida-persicifolia*) and one unresolved quartet (*calophylla-micrantha-multiflora-parviflora*). Successive reweighting produced, after three iterations, a single stable island of 100 trees, each 34.3 steps long, resolving 11 of 22 potential nodes within *Clermontia* (**Fig. 1B**). Seven characters were down-weighted in the latter analysis: petiole length, number of flowers per inflorescence, pedicel length, hypanthium shape, sepal texture, wine-colored corollas, and anther tube length. The strict consensus of the weighted analysis produced a tree that is slightly more resolved than that for the unweighted analysis, but which is otherwise consistent with the latter.

### DNA-based phylogenies

**Figure 2:**
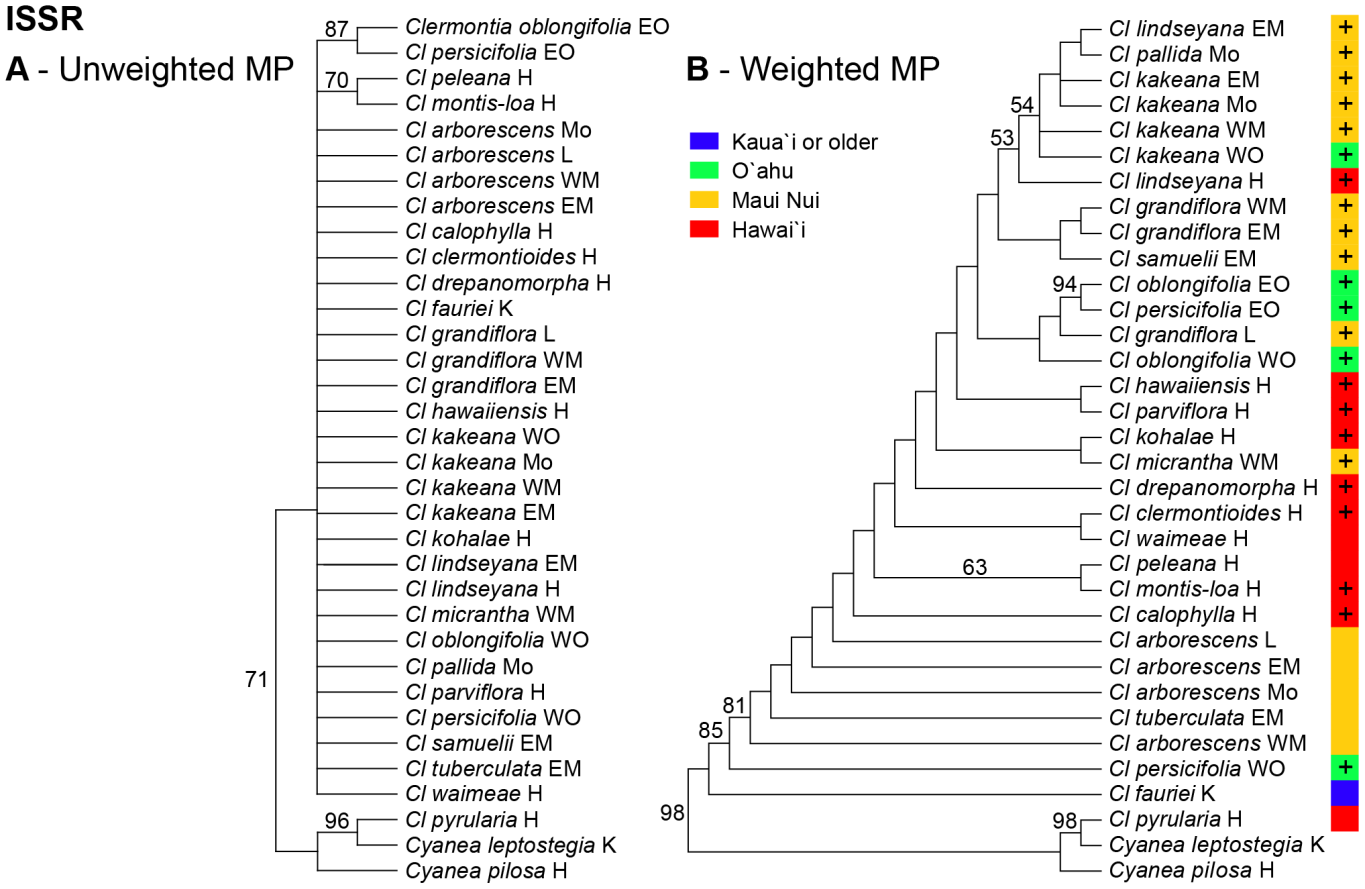
Phylogenetic relationships within *Clermontia* (32 populations spanning all 21 extant species, with two outgroup *Cyanea* species) based on 58 ISSR characters. Numbers above branches indicate bootstrap support levels ≥ 20%. (A) MP phylogeny based on unweighted characters. The tree shown is the strict consensus of 2296 shortest trees; (B) MP phylogeny based on sequential reweighting of ISSR characters; tree is strict consensus of six shortest trees. Colored band shows island distribution of populations (see key) and presence (+) vs. absence of petaloid sepals.

The nine ISSR primers yielded 58 bands, of which 3 were constant and 55 were phylogenetically informative. The initial heuristic search produced 19 islands with a total of 2296 most parsimonious trees, each of length 219 steps (CI  =  0.25; CI'  =  0.13). The strict consensus of these trees resolved only *peleana-montis-loa, oblongifolia-persicifolia,* and *pyrularia* sister to *Cy. leptostegia* within *Cyanea* (**Fig. 2A**). Two cycles of successive reweighting produced one stable island of six most parsimonious trees, each of length 55.0 steps (CI  =  0.41, CI'  =  0.28 excluding autapomorphies). Few polytomies remained in the strict consensus of these six trees, which placed *Cl. fauriei* sister to all other members of *Clermontia* proper, then *Cl. persicifolia* from West Òahu, then a grade consisting of *Cl. arborescens, Cl. tuberculata,* and *Cl. calophylla* (**Fig. 2B**). While the strict consensus of the weighted ISSR analysis is nearly completely resolved, most nodes are poorly supported, with a bootstrap support value < 50%.

**Figure 3:**
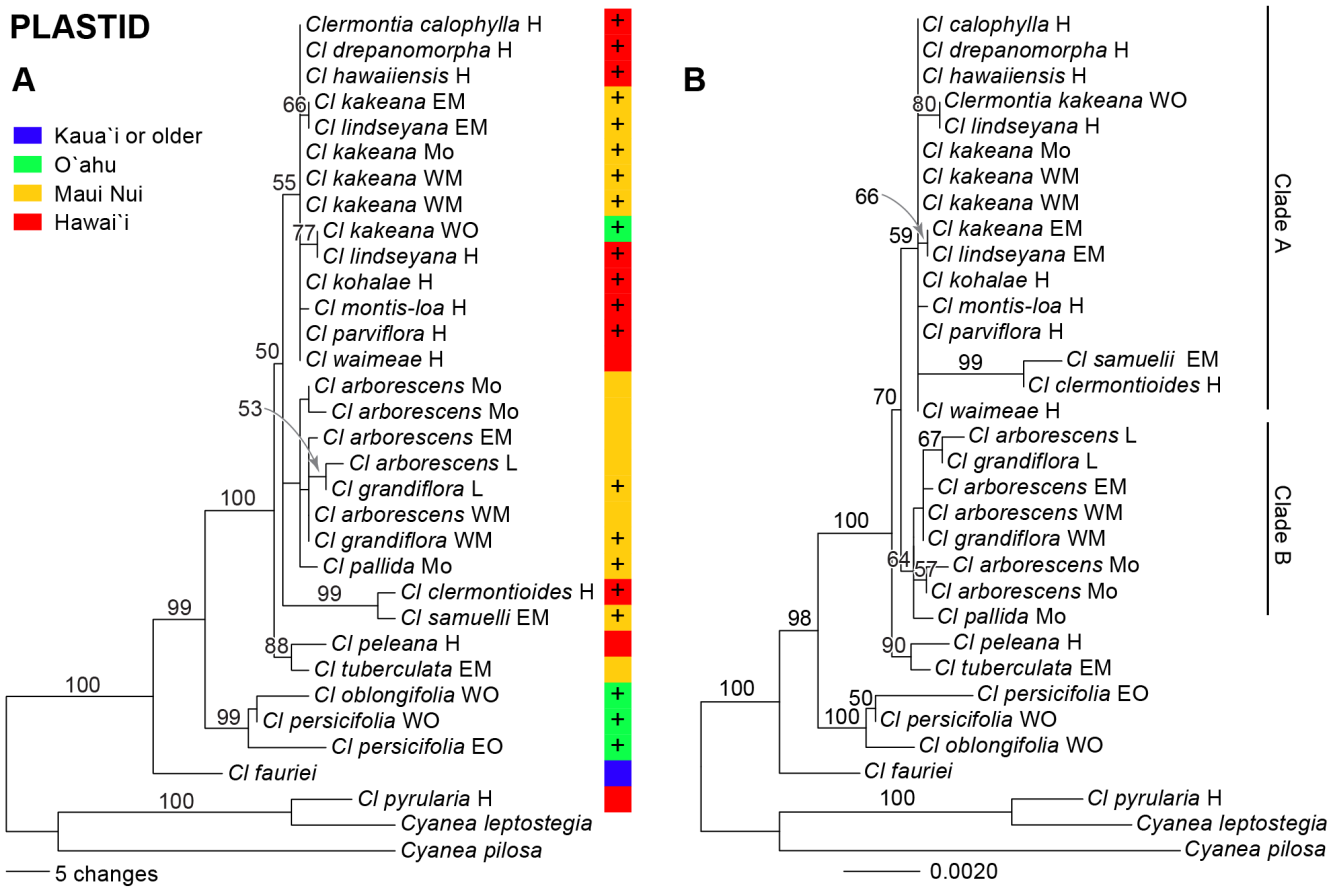
Phylogeny of ***Clermontia*** **(31 populations spanning all extant species except** ***Cl. micrantha***, with two outgroup *Cyanea* species) based on 4532 aligned nucleotides for five non-coding regions of the plastid genome. Branch lengths are proportional to inferred genetic changes down each branch. (A) MP phylogram consistent with the majority-rule consensus of 76 shortest trees based on plastid sequence data. Colored band shows island distribution of populations (see key) and presence (+) vs. absence of petaloid sepals. Numbers above branches indicate bootstrap support levels ≥ 20%. (B) ML phylogram based on plastid sequence data. Numbers above branches indicate bootstrap support levels ≥ 20%.

The five non-coding regions of plastid DNA produced 4532 aligned nucleotides, of which 4355 were constant, 98 were parsimony-uninformative, and 79 were parsimony-informative. A heuristic search produced one island of 76 shortest trees, each 194 steps long, with CI  =  0.94 and CI'  =  0.87 and a strict consensus tree with only 9 of 29 potential nodes within *Clermontia* resolved (tree not shown). In the plastid strict consensus, *Cl. fauriei* was sister to all *Clermontia* except *Cl. pyrularia* (which itself is sister to *Cyanea leptostegia* within *Cyanea*), and *Cl. persicifolia-oblongifolia* was sister to a largely unresolved clade including all other species sequenced. The plastid majority-rule tree, by comparison, had substantially greater structure, with *peleana*-*tuberculata* sister to other elements of the large, mostly unresolved clade in the strict consensus, and those other elements being divided into three weakly supported clades (**Fig. 3A**). The ML tree placed *peleana-tuberculata* sister to the same large clade, and divided the latter into two weakly supported subclades, with clade A including *Cl. pallida* and all populations of *Cl. arborescens* and *Cl grandiflora,* and clade B containing all remaining species, with all species except *Cl. clermontioides* and *Cl. samuelii* showing little or no molecular divergence from each other (**Fig. 3B**). This topology is essentially identical to the MP majority-rule consensus, except that *clermontioides-samuelii* moved from clade B in the ML tree to an unresolved trichotomy involving it, clade A, and the little-divergent remainder of clade B (*Cl. calophylla, drepanomorpha, hawaiiensis, kakeana, kohalae, lindseyana, montis-loa, parviflora, waimeae*). Note that *clermontioides-samuelii* sits on an incongruously long branch in the ML tree. The BI tree (not shown) had a topology identical to the ML tree.

The ILD test revealed highly significant (P<0.001) incongruence in phylogenetic structure between the largely nuclear ISSR data and the plastid sequence data. Moderately (≥ 70% BS) to strongly (≥ 90% BS) supported conflicts in position between the ISSR and plastid trees occur in several taxa, including (1) *Cl. persicifolia*-*Cl. oblongifolia* from East Òahu and *Cl. oblongifolia* from West Òahu; (2) *Cl. tuberculata* from East Maui; and (3) *Cl. peleana* from Hawaìi (**Figs. 2** **and** **3**). We also removed (4) *Cl. pyrularia* from Hawaìi, based on the conflict in its position in *Clermontia* based on morphology and in *Cyanea* based on molecular data. Removing these taxa left a substantial conflict between the ISSR and plastid trees in the position of *Cl. samuelii,* with that species sister to *Cl. clermontioides* with 93% BS in the plastid tree, and sister to populations of *Cl. grandiflora* in the ISSR tree (trees not shown). We therefore also pruned *Cl. samuelii* from the data set; a final ILD test show no significant incongruence between the ISSR and plastid data for the remaining 24 taxa of *Clermontia* and two of *Cyanea* (P>0.21).

**Figure 4:**
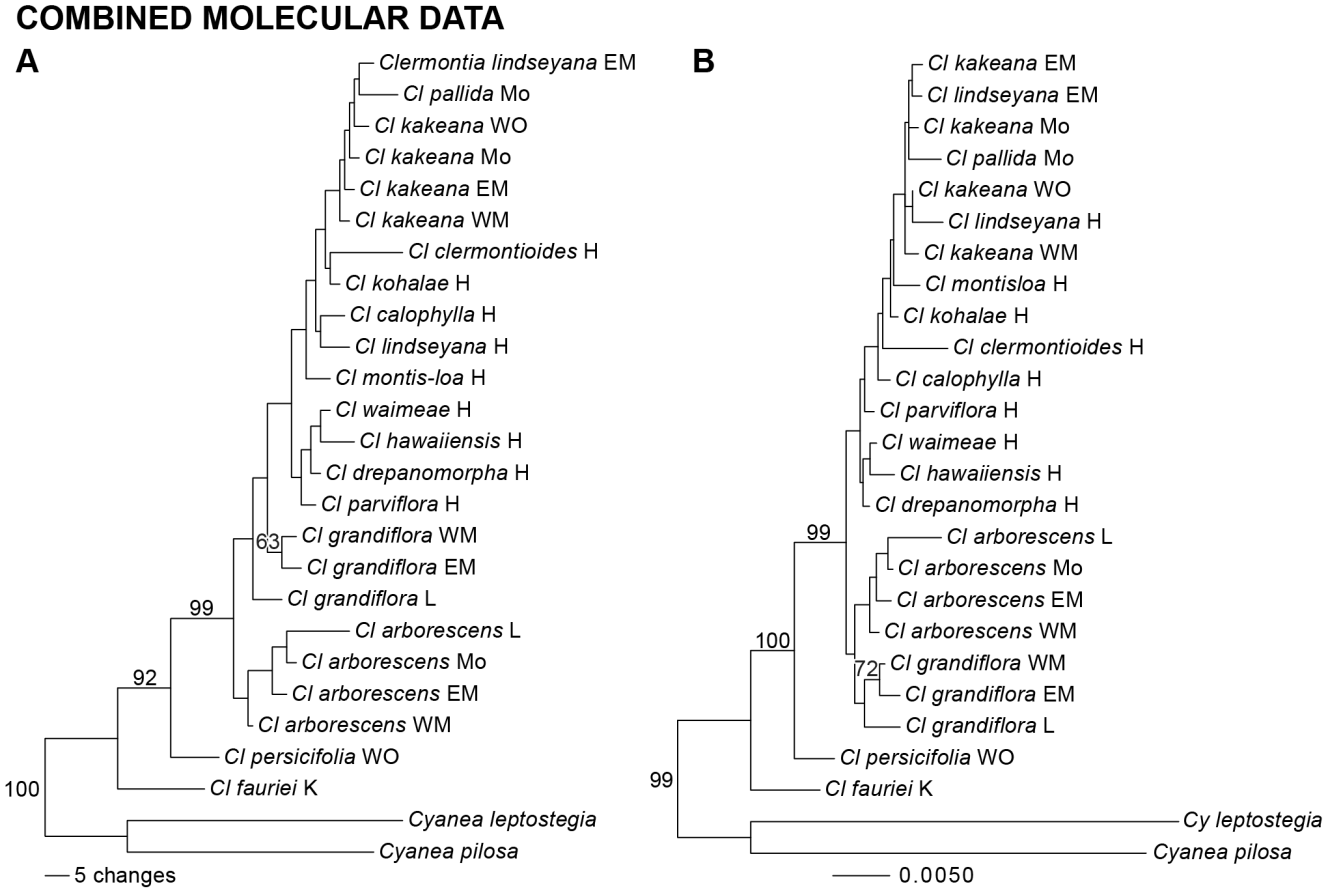
Phylogeny of *Clermontia* (24 populations spanning 15 apparently non-reticulate species, with two outgroup *Cyanea* species) based on the combined molecular data. Branch lengths are proportion to inferred genetic changes down each branch. (A) MP phylogram of the single shortest tree; numbers above branches indicate bootstrap support levels ≥ 20%. (B) ML phylogram; numbers above branches indicate bootstrap support levels ≥ 20%. Colored band shows island distribution of populations (see key) and presence (+) vs. absence of petaloid sepals.

The combined ISSR and plastid sequence data included 86 parsimony-informative characters and produced one shortest MP tree, 339 steps long with CI  =  0.61 and CI'  =  0.40 (**Fig. 4A**). In this tree, *Cl. fauriei* was sister to all other *Clermontia,* with *Cl. persicifolia,* a monophyletic *Cl. arborescens,* and a paraphyletic *Cl. grandiflora* sister to progressively more inclusive, nested clades. The innermost clade included a grade of nine species from Hawaìi, in which was embedded a complex involving *Cl. kakeana, Cl. lindseyana,* and *Cl. pallida,* almost exclusively from Maui Nui; branches within this innermost clade are all short and weakly supported. The MP tree based on the combined molecular data strongly supported the monophyly of the non-reticulate taxa of *Clermontia* and the clade sister to *Cl. persicifolia,* as well as the positions of *Cl. fauriei* and *Cl. persicifolia*. The ML tree based on the combined data (**Fig. 4B**) was quite similar to the MP tree, but (with weak support) identified *Cl. grandiflora* as being monophyletic and placed it sister to *Cl. arborescens.* In addition, ML placed *Cl. lindseyana* from Hawaìi into the *kakeana-lindseyana-pallida* complex from Maui Nui. Finally, BI produced a tree (not shown) quite similar to the ML tree. The most notable differences between the BI and ML trees are (1) the shift of *pallida* sister to the *arborescens-grandiflora* clade, and (2) the relatively high posterior support values for the *pallida-arborescens-grandiflora* clade, its sister clade, and the *kakeana-lindseyana* clade.

### Historical biogeography

**Figure 5:**
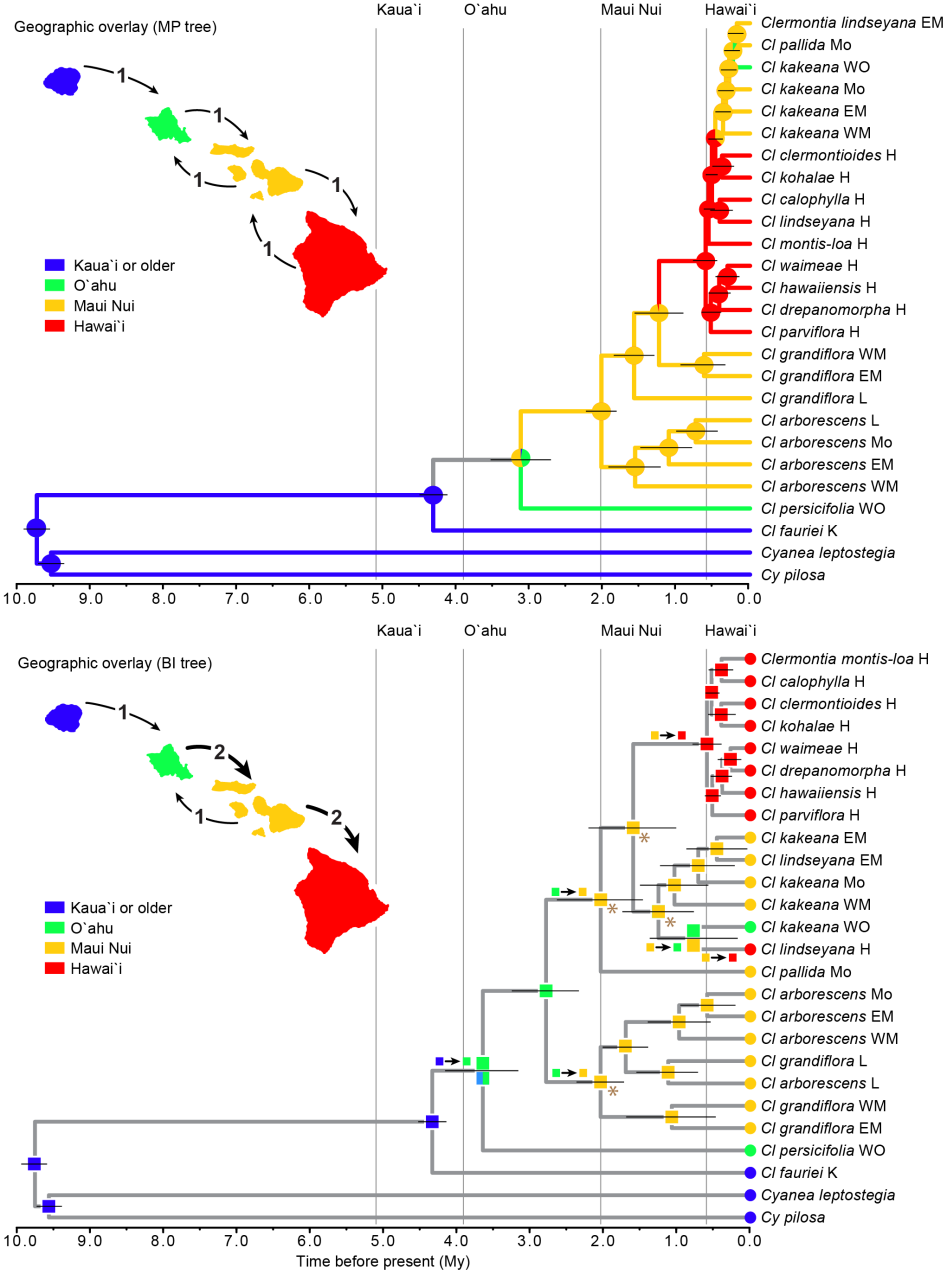
Overlay of geographic distribution of *Clermontia* on chronograms based on the combined molecular data. Inset maps shows the inferred number of inter-island dispersal events needed to account for the distribution of present-day non-reticulate taxa based on the MP reconstruction on the MP and BI trees, respectively, using delayed transformation. (A) Shifts in insular distribution plotted on the MP chronogram; branch colors represent inferred ancestral states based on parsimony mapping. Grey represents uncertainty. Pie diagrams represent inferred ancestral states under Bayesian inference. Horizontal razor lines represent the 95% confidence intervals about each date.. (B) Shifts in distribution plotted on the BI chronogram.

In the MP combined molecular phylogeny, inter-island dispersal events among non-reticulate taxa appeared to be initially consistent with the progression rule under parsimony mapping, but with later reversal on a few short, weakly supported branches (**Fig. 5A**). *Clermontia fauriei* from Kauài diverged from the deepest node; *Cl. persicifolia* from Òahu, from the next shallowest node; and *Cl. arborescens* and then *Cl. grandiflora* from Maui Nui, from the next several shallowest nodes. Dispersal to Hawaìi was inferred for the clade sister to *Cl. grandiflora* from East and West Maui, with back-dispersal to Maui Nui by the large *kakeana-lindseyana-pallida* clade, and back-dispersal to O'ahu in one population of *Cl. kakeana* (**Fig. 5A**).

Essentially the same pattern was reconstructed using BEAST, with an Òahu-Maui Nui uncertainty at the node joining *Cl. persicifolia* to its sister clade (**Fig. 5B**). The estimated age of the initial dispersal to O'ahu was ca. 3.9 Mya; the initial dispersal to Maui Nui, ca. 2.0 Mya; and the initial dispersal to Hawaìi, ca. 0.6 Mya. The latter two dates reflect constraints imposed by the estimated ages of Maui Nui and Hawaìi; a BEAST analysis without those constraints (not shown) implied initial arrival on Maui Nui ca. 3.9 Mya and initial arrival on Hawaìi ca. 2.0 Mya. A DEC analysis implied dispersal from an older island to Kauài ca. 4.4 Mya; dispersal from Kauài to O'ahu ca. 3.9 Mya; two independent dispersal events from O'ahu to Maui Nui ca. 2.0 Mya; one dispersal event from Maui Nui to Hawaìi ca. 0.6 Mya, with a second sometime durng the last 0.6 My; and one back-dispersal from Maui Nui to O'ahu ca. 0.8 Mya (**Fig. 5B**). The four nodes marked with an asterisk in **Fig. 5B** actually were not resolved as being Maui Nui vs. a 50∶50 chance of O'ahu vs. Maui Nui in the most likely analysis, but the simpler form shown (where all four nodes were resolved as Maui Nui) did not differ significantly from the more complex resolution.

### Petaloid sepal character-state reconstruction

**Figure 6:**
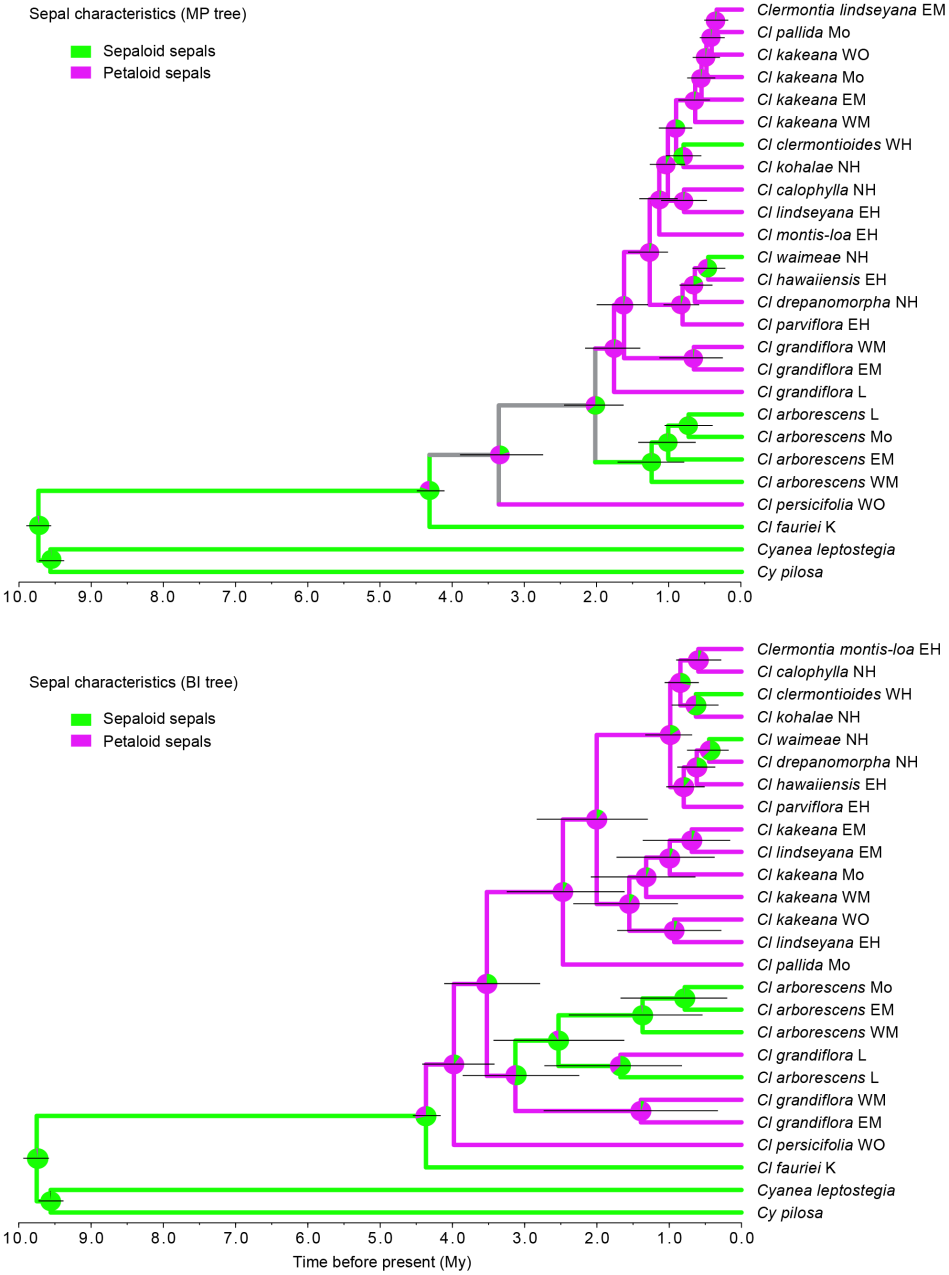
Overlay of petaloid vs. **sepaloid sepals on chronograms based on the combined molecular data.** (A) Shifts in sepal nature plotted on the MP chronogram; branch colors represent inferred ancestral states based on parsimony mapping. Grey represents uncertainty. Pie diagrams represent inferred ancestral states under Bayesian inference. Horizontal razor lines represent the 95% confidence intervals about each date. (B) Shifts in sepal nature plotted on the BI chronogram.

Parsimony mapping of sepal characteristics onto the MP combined molecular phylogeny implies that petaloid sepals arose twice, in *Cl. persicifolia* on Òahu by 3.3 Mya, and in *Cl. grandiflora* – and the taxa it subtends – on Maui Nui by 2.0 Mya (**Fig. 6A**). Petaloid sepals appear to have been lost independently on Hawaìi twice, in *Cl. clermontioides* by 0.9 Mya and in *Cl. waimeae* by 0.5 Mya. Bayesian mapping implies essentially the same pattern (**Fig. 6A**). Alternatively, and perhaps more plausibly, the conflict between the ISSR and plastid trees and the crossing it implies between *Cl. grandiflora* and *Cl. oblongifolia*, and between *Cl. oblongifolia* and *Cl. persicifolia*, suggests that petaloid sepals may have arisen only once, in the ancestor of *Cl. grandiflora* and the taxa the latter subtends, with petaloid sepals occurring in *Cl. persicifolia* via introgression from *Cl. grandiflora* and *Cl. oblongifolia.*

Parsimony mapping onto the BI combined tree implied two independent origins of petaloid sepals, on Òahu no later than 3.9 Mya in the clade subtended by *Cl. persicifolia,* and on Lanài no later than 1.6 Mya in *Cl. grandiflora* (**Fig. 6B**). This mapping implies three secondary losses of petaloid sepals, in *Cl. arborescens* on Maui Nui no later than 2.4 Mya, in *Cl. clermontioides* on Hawaìi no later than 0.8 Mya, and in *Cl. waimeae* on Hawaìi no later than 0.5 Mya. Bayesian mapping yields essentially the same pattern. Again, a more plausible scenario might involve an origin of petaloid sepals in *Cl. persicifolia* via introgression. Overall, the scenario for the evolutionary gain and loss of petaloid sepals appears to be simplest if we assume the MP combined molecular phylogeny and the introgressive origin of such sepals in *Cl. persicifolia*.

## Discussion

### Species relationships and apparent patterns of hybridization/introgression

Neither morphology, nor ISSR variation, nor plastid sequences, nor the combined molecular data supported the monophyly of *Clermontia* sect. *Clermontioides,* defined by a lack of petaloid sepals, or of *Clermontia* sect. *Clermontia,* marked by their presence. Unweighted morphological data resolved only four of 22 possible nodes within *Clermontia* under maximum parsimony; sequential reweighting resolved 11 nodes in the MP strict consensus tree (**Fig. 1A,B**). Both analyses supported the monophyly of only three of the six series described by Lammers [5] based on his cladistic analysis of morphology, including ***Unilabiatae*** (*Cl. fauriei, Cl. peleana*), ***Sarcanthae*** (*Cl. arborescens, Cl. tuberculata*), and ***Parviflorae*** (*Cl. calophylla, Cl. micrantha, Cl. multiflora, Cl. parviflora*) (**Fig. 1**).

None of the morphological series proposed by Lammers [5] was supported as monophyletic by the ISSR, plastid, or combined molecular phylogenies (**Figs. 2** **,** **3**). The closest approach to support of a series was the identification of a grade including *Cl. arborescens, Cl. tuberculata,* and *Cl. calophylla* in the ISSR tree, approaching series *Sarcanthae*; and Clade A in the ML and BI plastid trees, approaching the large series *Kakeanae* but including *Cl. calophylla* (excluded by morphology by its small flowers), *Cl. hawaiiensis* (excluded based by its tubular corolla), and *Cl. clermontioides* and *Cl. waimeae* (excluded by lack of petaloid sepals), and excluding *Cl. persicifolia* (initially included based on petaloid sepals). Interestingly, the ISSR data are *not* consistent with the monophyly of any individual species sampled more than once (*Cl. grandiflora, Cl. kakeana, Cl. oblongifolia, Cl. persicifolia*), while the plastid sequence data are consistent with monophyly in each of these cases but fail to demonstrate it positively in any one (**Figs. 2** **,** **3**). The MP combined molecular tree identified *Cl. arborescens* as monophyletic, *Cl. grandiflora* and *Cl. kakeana* as paraphyletic, and *Cl. lindseyana* as polyphyletic (**Fig. 4A**). The ML and BI combined molecular trees fail even to resolve *Cl. arborescens* as monophyletic (**Fig 4B**).

The ISSR and plastid sequence data showed a highly significant degree of incongruence, with conflict between the strict consensus trees suggesting several instances of hybridization, introgression, or incomplete lineage sorting, involving the five species *Cl. persicifolia, Cl. oblongifolia, Cl. tuberculata, Cl. peleana,* and *Cl. samuelii.* Conflict between morphology and molecular data also point to intergeneric hybridization in *Cl. pyrularia* and *Cl. tuberculata.* Given that several previous authors have made compelling cases for crossing between species of *Clermontia* [1], [2], [5], [6], [7], we believe that these taxa are likely to be products of recent hybridization and/or introgression, as detailed here:

1. We propose that *Clermontia pyrularia* is an intergeneric hybrid, involving a cross between a species of the purple-fruited clade of *Cyanea* and *Clermontia clermontioides* subsp. *clermontioides. Clermontia pyrularia* was strongly supported as closely related to *Cy. leptostegia*, a representative of the purple-fruited clade, in both the ISSR and plastid sequence trees. Morphologically, however, *Cl. pyrularia* it is most closely similar to *Cl. clermontioides* subsp. *clermontioides*, with which it is known to co-occur on leeward Hualalai and Mauna Loa [5]. The few remaining individuals of *Cyanea hamatiflora* subsp. *carlsonii* – one of two species of purple-fruited *Cyanea* to grow on the Big Island – co-occur with *Cl. clermontioides* subsp. *clermontioides* [5, 7; TJ Givnish and KJ Sytsma, pers. obs.]. Extinct, purple-fruited *Cy. giffardii* from one kipuka on windward Mauna Loa was, by contrast, disjunct from *Cl. clermontioides* subsp. *clermontioides* [5], [7].
2. We propose that *Cl. oblongifolia* is a hybrid involving *Cl. persicifolia* and *Cl. grandiflora,* based on the disparate positions of *oblongifolia* in the ISSR and plastid trees. Lammers [5] noted that *Cl. oblongifolia* is similar vegetatively to *Cl. persicifolia,* and similar in many floral traits to *Cl. grandifolia. Cl. grandifolia* and *Cl. oblongifolia* share the unusual characteristic of pendent flowers. In addition, *Cl. oblongifolia* is the most widespread species of *Clermontia*, occurring on four islands, and presumably is highly mobile. A cross with *Cl. grandifolia* may have introduced the gene(s) controlling sepaloid petals into *Cl. oblongifolia,* and introgression from *Cl. oblongifolia* into *Cl. persicifolia* may have transferred the gene(s) into the latter as well, which would account for what would otherwise be an extraordinary additional origin of petaloid sepals in *Cl. persicifolia.* A careful analysis of *PISTILLATA* sequences [18] might be used to test this hypothesis.
3. *Clermontia tuberculata* may represent the product of intergeneric hybridization involving *Cl. arborescens* and *Cyanea aculeatiflora.* The ISSR data clearly identify *Cl. tuberculata* as part of the *Cl. arborescens* complex, while the plastid data (**Fig. 3**) place *Cl. tuberculata* closest to *Cl. peleana*. Morphologically, *Cl. tuberculata* is most like *Cl. arborescens*, and occurs in and near populations of *Cl. arborescens* subsp. *waihiae* on East Maui [5]. However, unlike any other member of *Clermontia* – or, indeed, almost any other angiosperm species – *Cl. tuberculata* has prickly (muricate) inflorescence axes, sepals, and petals [5]. *Clermontia tuberculata* is wholly sympatric with *Cyanea aculeatiflora,* with which it shares the highly unusual trait of muricate flowers. *Cyanea aculeatiflora,* in turn, is partly sympatric with *Cy. macrostegia* ssp. *macrostegia,* which lacks muricate flowers but is otherwise similar to *Cy. aculeatiflora.* Lammers [5] suggested that hybridization/introgression may have transferred muricate flowers into *Cl. tuberculata* from populations of *Cy. aculeatiflora* that, in turn, were recently derived from *Cy. macrostegia.* We do not yet have molecular data from *Cyanea* to test this hypothesis, however. Convergence seems far less likely an explanation for the muricate inflorescence axes, sepals, and petals of *Cl. tuberculata.*
4. We propose that *Clermontia peleana* is the result of hybridization involving a member of the *arborescens-tuberculata* complex (with which it is associated in the plastid tree) and *Cl. montis-loa* (to which it is sister in the ISSR tree) (**Figs. 2** **,** **3**). In fact, *Cl. peleana* is sister to *Cl. tuberculata* itself in the plastid tree, but it lacks the distinctive muricate flowers of *Cl. tuberculata,* suggesting that a close relative within the *arborescens-tuberculata* complex may have been the ancestor. Prior to 1930, *Cl. peleana* subsp. *singuliflora* grew on East Maui, home to *Cl. tuberculata* and one of the latter's inferred ancestors, *Cl. arborescens* subsp. *waihiae* (see above). *Clermontia peleana* subsp. *singuliflora* also was native to windward Mauna Kea on Hawaìi, and in 2010 was rediscovered in the Kohala Mountains; its former haunts on Mauna Kea lie only 8 km from the nominate subspecies of *Cl. peleana* and *Cl. montis-loa* in windward Hawaìi [see 5]. It therefore seems likely that *Cl. peleana* arose via hybridization and/or introgression of *Cl. arborescens* subsp. *waihiae* and *Cl. montis-loa* on East Maui and/or northern Hawaìi.

This scenario may help explain the origin of wine-colored corollas in *Cl. peleana* subsp. *peleana* (which shares this trait with *Cl. montis-loa*), and the association of *Cl. fauriei* and *Cl. peleana* and of *Cl. arborescens* and *Cl. tuberculata* in the morphology-based trees (**Fig. 1A,B**) and of *Cl. arborescens* and *Cl. tuberculata* in the weighted ISSR tree. All four species share fleshy corollas, consistent with *Cl. Faurń ei* and *Cl. arborescens* emerging from two of the three deepest nodes in *Clermontia,* and with *Cl. peleana* and *Cl. tuberculata* emerg ning from *Cl. arborescens* via hybridization/introgression. Unilabiate corollas are unique to *Cl. fauriei* and *Cl. peleana* within the genus, separate from the bilabiate *Cl. arborescens* and *Cl. tuberculata. Clermontia* must have undergone at least two transitions in floral zygomorphy, given that it includes taxa with unilabiate, bilabiate, and rotate (nearly actinomorphic) corollas. Its sister genus *Cyanea*, like most members of Lobeliaceae, generally have bilabiate corollas, but one group of palmiform species also evolved unilabiate corollas, as did distantly related *Lobelia* sect. *Tupa* from South America, sect. *Tylomium* from the Greater Antilles, sect. *Heteroclita* from South America, and sect. *Jasionopsis* from South Africa, while closely related Hawaiian genus *Brighamia* has corollas that are nearly actinomorphic (see phylogenies and character-state descriptions in [**5]**, [14], [67], [68****]). The pattern of zygomorphy, in other words, shows substantial evolutionary lability across lobeliads as a whole, although a broader and more complete phylogeny will be needed to analyze the precise number of independent origins of different zygomorphic patterns.

1. We propose that *Cl. samuelii* is the result of hybridization between *Cl. grandiflora* (with which it is associated in the ISSR tree) and *Cl. clermontioides* (with which it is associated in the plastid tree, and in the initially pruned MP tree based on the combined molecular data). *Clermontia samuelii* has flowers quite similar in shape to those of *Cl. grandiflora,* but smaller; its range immediately abuts that of *Cl. grandiflora* subsp. *munroi* in easternmost East Maui, and lies across a relatively short distance of ocean from the western edge of the distribution of *Cl. clermontioides* subsp. *clermontioides* in western Hawaìi [5].
2. Finally, frequent hybrids involving *Cl. calophylla, Cl. drepanomorpha, Cl. hawaiiensis, Cl. kohalae, Cl. montis-loa,* and *Cl. parviflora* on Hawaìi may reflect their very close relationship to each other in Clade A of the ML and BI plastid trees. The apparent lack of hybridization involving the other taxa in Clade A may reflect largely non-overlapping distributions on Maui Nui of closely related *Cl. kakeana* and *Cl. lindseyana*, and largely non-overlapping distributions on Hawaìi of *Cl. clermontioides* (mostly leeward Hawaìi) and *Cl. waimeae* (mostly lower elevations in the Kohala Mountains in ne Hawaìi).

### Historical biogeography

Both phylogenies based on the combined molecular data, pruned of apparently reticulate taxa, provide substantial support for the progression rule. Contrary to Lammers [6], our MP analysis places the origin of *Clermontia* on Kauài or an older island, with *Cl. fauriei* (the only species native to Kauài, but once also found on the older, western side of the next younger island, Òahu) emerging from the oldest node, *Cl. persicifolia* (the only species restricted to Òahu) emerging from the next oldest node, then *Cl. arborescens* and *Cl. grandiflora* from Maui Nui at successively younger nodes, then a large grade endemic to Hawaìi, and finally back-dispersal to Maui Nui and then Òahu implied by inclusion of the *Cl. kakeana-lindseyana-pallida* complex (**Fig. 5A**). The current distribution of species and the MP analysis based on the combined molecular data are not adequate, however, to conclude with certainty that *Cl. persicifolia* resulted via dispersal from Kauài to Òahu.

The estimated dates of initial dispersal from one island to the next youngest in the chain based on the MP chronogram are largely consistent with the known ages of each target group of tall islands: 3.3 Mya for dispersal to Òahu, vs. its maximum age of 3.9 Mya; and 2.0 Mya for dispersal to Maui Nui, vs. its maximum age of 2.2 Mya (**Fig. 5A**). However, the estimated date of initial dispersal to Hawaìi of 1.4 Mya substantially antedates the emergence of the youngest island not earlier than 0.6 Mya. Our DEC analysis eliminates this conflict, placing the initial arrival of *Clermontia* on Hawaìi from Maui Nui at 0.6 Mya, and a subsequent independent arrival of *Cl. lindseyana* on Hawaìi from Maui Nui sometime in the last 0.6 My (**Fig. 5B**). DEC places the arrival of *Clermontia* on Kauài at ca. 4.4 Mya; DEC leaves the stem node of *Cl. persicifolia* equivocal as O'ahu or Kauài/O'ahu, but nevertheless clearly implies one dispersal event from Kauài to O'ahu. Overall, DEC implies one dispersal from Kauài to O'ahu; two from O'ahu to Maui Nui; two from Maui Nui to Hawai'i; and one back-dispersal from Maui to O'ahu (**Fig. 5B**). Thus, five of six inter-island dispersal events implied by DEC analysis for non-reticulate taxa are consistent with the progression rule, compared with three of five such events implied by MP analysis. By contrast, Lammers [5] used his morphology-based analysis to infer 12 inter-island dispersal events, only five of which were consistent with the progression rule.

**Figure 7:**
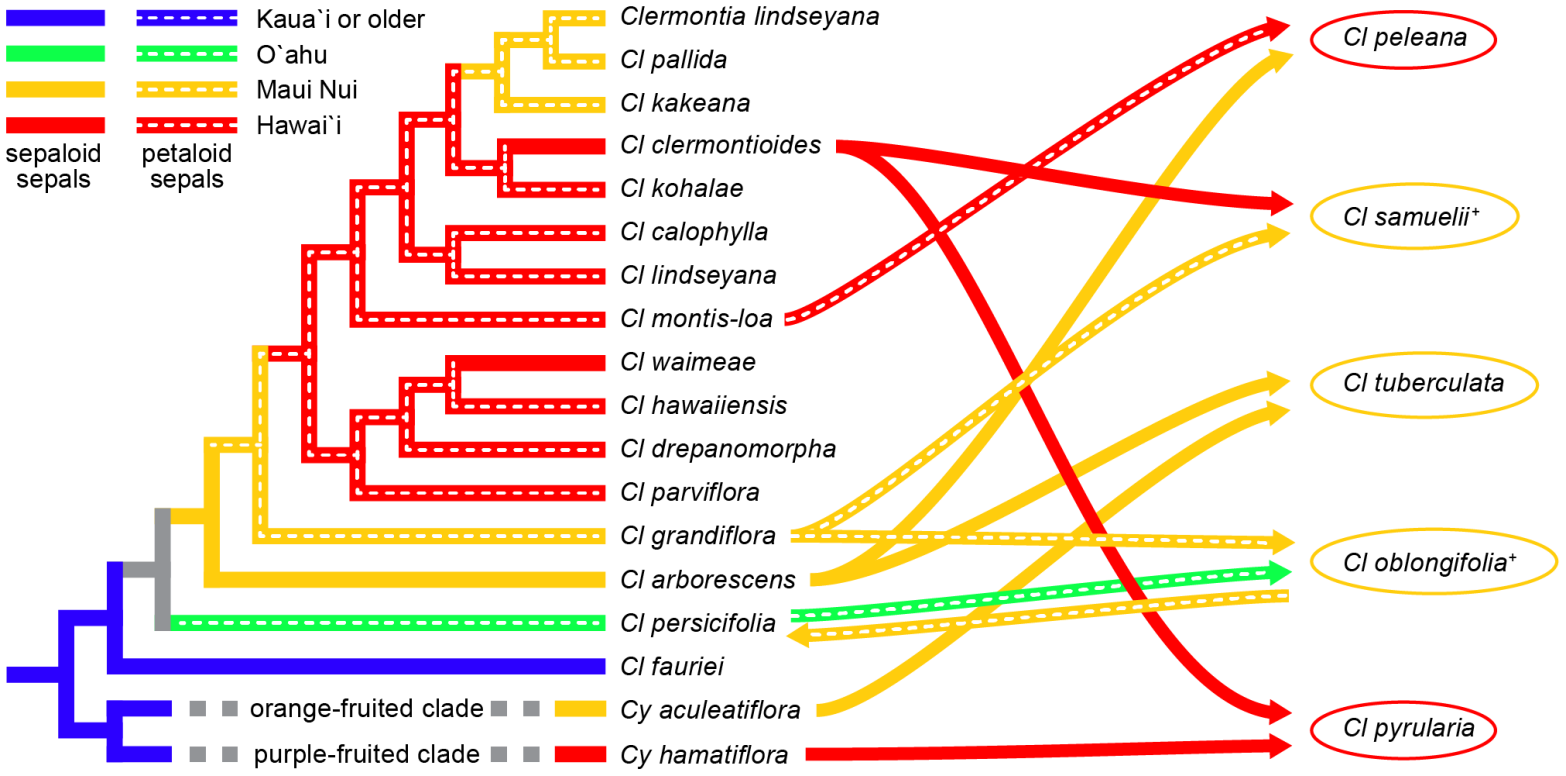
Summary of patterns of geographic dispersal, sepal evolution, and reticulate speciation in *Clermontia* inferred from ISSR, plastid, and combined molecular data and morphological variation. Cladogram illustrates phylogenetic relationships among non-reticulate taxa based on MP. Branch color represents inferred ancestral distribution under parsimony; presence/absence of dashed line represents presence/absence of petaloid sepals. Curved arrows, colors, and dashed lines leading to the right represent apparent hybrid/introgressive origins of five reticulate taxa and the distribution of parental taxa. Superscript +'s on the latter indicate the presence of petaloid sepals. Curved arrow leading to the left represents apparent introgression of petaloid sepals into *Cl. persicifolia* from *Cl. oblongifolia* (and, ultimately, from *Cl. grandiflora*). The basal split of *Cyanea* into orange- and purple-fruited clades is shown, together with the particular species of *Cyanea* thought to be involved in the origins of *Cl. pyrularia* and *Cl. tuberculata* based on morphology and/or geographic distribution.

Proposed reticulation events suggest, in addition, one dispersal event from Òahu to Maui Nui and a return flight from Maui Nui to Òahu to account for dispersal of *Cl. oblongifolia* down the chain, and subsequent introgression of gene(s) derived from *Cl. grandiflora* into *Cl. persicifolia* on Òahu (**Fig. 7**). An additional dispersal from East Maui to Hawaìi is required to account for the origin of *Cl. peleana* via introgression between *Cl. arborescens* subsp. *waihiae* and *Cl. montis-loa.* Finally, back dispersal (or, at last, gene flow) of *Cl. clermontioides* from Hawaìi to East Maui is required for the origin of *Cl. samuelii* there via hybridization with *Cl. grandiflora* (**Fig. 7**). Overall, based on the DEC analysis, seven of the proposed inter-island dispersal events were down the chain, consistent with the progression rule, and three involved back dispersal up the chain. Most of the latter appear to have occurred relatively late in the evolution of *Clermontia,* with the possible exception of the (undated, unstudied) back-dispersal of introgressed *Cl. oblongifolia* from Maui Nui to Òahu. Based on MP, five of nine dispersal events were consistent with the progression rule. In both cases, early dispersal events appear to have been almost entirely consistent with the progression rule.

### Petaloid sepal character-state reconstruction

Based on the MP combined-data tree, the simplest scenario for the evolution of petaloid sepals is that they were initially absent in *Clermontia*, arose once in the clade subtended by *Cl. grandiflora,* spread into *Cl. oblongifolia* via introgression from *Cl. grandiflora,* and into *Cl. persicifolia* from *Cl. oblongifolia*, and with subsequent losses of such sepals in *Cl. waimeae* and *Cl. clermontioides.* Hybridization/introgression would account for the absence of petaloid sepals in *Cl. peleana* and *Cl. tuberculata* derived from *Cl. arborescens* bearing sepaloid sepals, crossed with *Cl. montis-loa* (which does have petaloid sepals) and *Cy. aculeatiflora* (which does not). This scenario is also largely consistent with those based on the ISSR or plastid data alone, but appears to be more plausible than either of them.

The ISSR tree implies that absence of petaloid sepals was the ancestral state, that there was one gain of petaloid sepals in the clade sister to *Cl. arborescens* from Molokài and East Maui, followed by three losses of such traits in *Cl. calophylla, Cl. peleana,* and *Cl. waimeae,* with introgression carrying the trait into *Cl. oblongifolia and C. persicifolia.* The latter, of course, is not based on the ISSR tree itself, but on the conflict between the ISSR and plastid trees, and the morphological similarities and distributions of *Cl. oblongifolia*, *Cl. persicifolia,* and *Cl. grandiflora.*

The plastid tree alone implies a much less plausible scenario, involving at least three independent gains of petaloid sepals and three losses. Given the obvious presence of reticulation in *Clermontia,* and the high likelihood that petaloid sepals involve a homeotic mutation in nuclear-encoded genes [18], it seems wiser to trace their evolution on the largely nuclear ISSR tree or – best – to trace it on the combined-data tree once the apparently reticulate taxa have been been pruned from the analysis.

Our results for the origin and spread of petaloid sepals, are consistent with but far more resolved than those presented by Hofer et al. [18] based on sequences of the non-transcribed spacer of 5S rDNA, and by Pillon et al. [19] based on SNPs. All four studies place *Cl. fauriei* sister to all other core *Clermontia*; Pillon et al. and our current paper identify *Cl. pyrularia* as falling within *Cyanea.* Our study differs from all previously published studies, however, in proposing that *Cy. pyrularia* is a hybrid including *Cl. clermontioides* and *Cy. hamatiflora* subsp. *carlsonii* of the purple-fruited clade of *Cyanea,* and in the details of relationships and at least six reticulation events within the rest of *Clermontia*, with important implications for the reconstruction of geographic spread and morphological evolution within the genus. Given the far greater number of informative characters in the combined molecular data set vs. morphology, the inability of the unweighted morphological data to recover almost any phylogenetic structure in *Clermontia*, and the power of the molecular data to pinpoint apparent hybridization/introgression events, we believe that the molecular data provide a much stronger basis for inferring phylogeny than do the morphological data.

Our current molecular data, however, are not adequate to fully resolve the phylogeny of *Clermontia* and clarify all questions regarding the diversification of this genus. Because we lack fully resolved, strongly supported trees based on plastid sequence data and multiple, individually powerful nuclear data sets, as well as coverage of several critical taxa in the sister genus *Cyanea,* we are not yet in a position to identify and unambiguously reconstruct many cladogenetic events and reticulations, or assemble the detailed history of geographic diversification and character evolution of *Clermontia*. As a result, we are uncertain whether we have been able to remove all reticulate taxa from our trees, or to identify all repeated layers of reticulation, and thus to derive a single, natural phylogeny based on combined nuclear and plastid data from which incongruence has been purged. Given the low levels of support and/or resolution in both our nuclear and plastid trees to date, it is clear that future progress on *Clermontia* evolution will require us to increase greatly the amount of data per taxon, to include multiple, individually highly informative nuclear loci, and to sample populations of each taxon more densely on each island on which it occurs. Given that the extant species of *Clermontia* have diverged from each other in less than 4.7 My, are known to cross with each other (and even with *Cyanea*) in several instances, and appear to be highly mobile within the Hawaiian archipelago, the requirement for greater density and extent of data to reconstruct evolution within *Clermontia* should come as no surprise. Such increases in data density per taxon and in taxon sampling will almost surely require using the power of next-generation sequencing, to which our labs are now turning.
